# Being part of change: partner perspectives on capacity building in a long-term community-engaged health equity initiative

**DOI:** 10.3389/fpubh.2025.1633625

**Published:** 2026-01-29

**Authors:** Celeste Nicholas, Tess D. Weathers, McKenzie Altman, Teddrick Hardy, Lisa K. Staten

**Affiliations:** 1 Department of Community and Global Health, Richard M. Fairbanks School of Public Health, Indiana University Indianapolis, Indianapolis, IN, United States; 2 No Days Off 317, Indianapolis, IN, United States

**Keywords:** capacity building, chronic disease prevention, collective impact, community engagement, diabetes prevention, health equity, multi-sector partnership

## Abstract

To address health disparities, community engagement (CE) approaches meaningfully and actively partner with affected communities for the long term. While the ultimate goal of CE is to affect population health, intermediate outcomes, like capacity building, can be used to assess progress toward achieving and sustaining health aims. The Diabetes Impact Project – Indianapolis Neighborhoods (DIP-IN) is a CE initiative aimed at reducing diabetes disparities through multi-sector collaboration and resident-driven decision-making. This study evaluates capacity building in DIP-IN through qualitative interviews with 28 partners, including residents, project staff, and organizational leaders. Partners across roles reported increased capacity, including enhanced leadership, professional development, improved data practices, the development of staffing structures, and increased investment in community health. Facilitators included DIP-IN CE principles of long-term commitment, respect, valuing resident expertise, and transparency. Slow progress toward goals was a main barrier. Findings suggest that the project is progressing towards ultimate aims around health equity, with the potential for long-lasting impacts. The study underscores the importance of sustained, resident-driven CE supported by formal structures like steering committees. Further, it demonstrates that capacity building is a critical indicator of progress toward achieving and sustaining health improvement goals. Practical tools are provided to support robust evaluation of capacity building in a variety of settings.

## Introduction

Many under-resourced and marginalized geographic communities experience significant health inequities that have been generations in the making. Community engagement (CE) approaches that meaningfully and actively involve residents of affected communities and organizations that serve them are widely recognized as essential for addressing these health inequities (1). *Principles of Community Engagement* (2025) defines CE as building “sustainable relationships through trust and collaboration, strengthening community well-being. The process should be enduring, equitable, and culturally sensitive to all participants with a shared goal of addressing the concerns of the community” (2). In addition to meeting basic ethical imperatives, CE is widely thought to make interventions more effective (3–6). CE informs context-specific solutions which have greater community ownership (2, 7) and builds capacity within the community to sustain long-term action on generational health disparities (8–13). Evaluating intermediate outcomes such as capacity building and empowerment are therefore critical in understanding how CE contributes to overall intervention effectiveness (12, 14–19).

The Diabetes Impact Project – Indianapolis Neighborhoods (DIP-IN) is an initiative focused on improving the health and well-being of three racially/ethnically diverse clusters of neighborhoods in Indianapolis that experience a disproportionately high burden of diabetes. At the start of the project, the estimated rate of diagnosed diabetes in DIP-IN areas was almost double the US rate (20, 21). CE is the foundation of DIP-IN and guides everyday implementation in multiple ways through a CE approach that emphasizes bi-directional leadership and community ownership (11, 22, 23). DIP-IN brings communities and multi-sector partners together to address diabetes disparities through intervention components that span the prevention continuum (24). Partners include community residents and local stakeholders from academia, city government, community development, community-based organizations, county health department, health industry, and the public hospital system. Core intervention components include neighborhood and health system-based community health workers, resident steering committees, and community health improvement projects (CHIPs) (24–29).

DIP-IN is a rare health equity initiative due to its long duration and high degree of deferral to residents in decision-making. Given our partner communities may have experienced disempowering, distrustful relationships with previous external initiatives, our CE approach focuses on building trust by shifting power from institutions to communities. This kind of approach, more than others (e.g., input, collaboration), is thought to support community ownership, “unlocking collective power and capacity for transformative solutions” (11). Capacity-building is assessed as an intermediate outcome that is a necessary step to achieving three main health aims that are focused on primary, secondary, and tertiary prevention of diabetes. We conceptualize capacity building in a broad sense to include processes and activities conducted by DIP-IN that enhance our ability to progress toward these health aims, sustain impact, and/or leverage action on health aims beyond diabetes. More specifically, capacity building occurs by strengthening the skills, resources and systems involved at individual, organizational, or community levels. Although capacity building in CE often refers to the community (30), we include all DIP-IN partners to provide a more comprehensive understanding of short and long-term collective impact.

Despite the prominence of capacity building in CE frameworks, there is a need for more research on how capacity building contributes to overall intervention effectiveness, particularly within initiatives of this scale, complexity, and duration (12, 14–19). This study addresses that gap by examining capacity building in processes and outcomes in DIP-IN through the perspectives of partners, including residents. We address key questions including: Is there evidence that being a DIP-IN partner builds capacity? How has DIP-IN’s CE approach contributed to increased capacity among partners? Throughout the analysis, we attend to perceived facilitators and barriers to capacity building. Findings advance scholarship centering partners’ perspectives on capacity building and which characteristics of CE they consider most influential (31–35). We also provide practical tools for evaluating capacity building including a conceptual model, interview guide, and outcomes table.

## Methods

### DIP-IN community engagement approach

**Figure 1:**
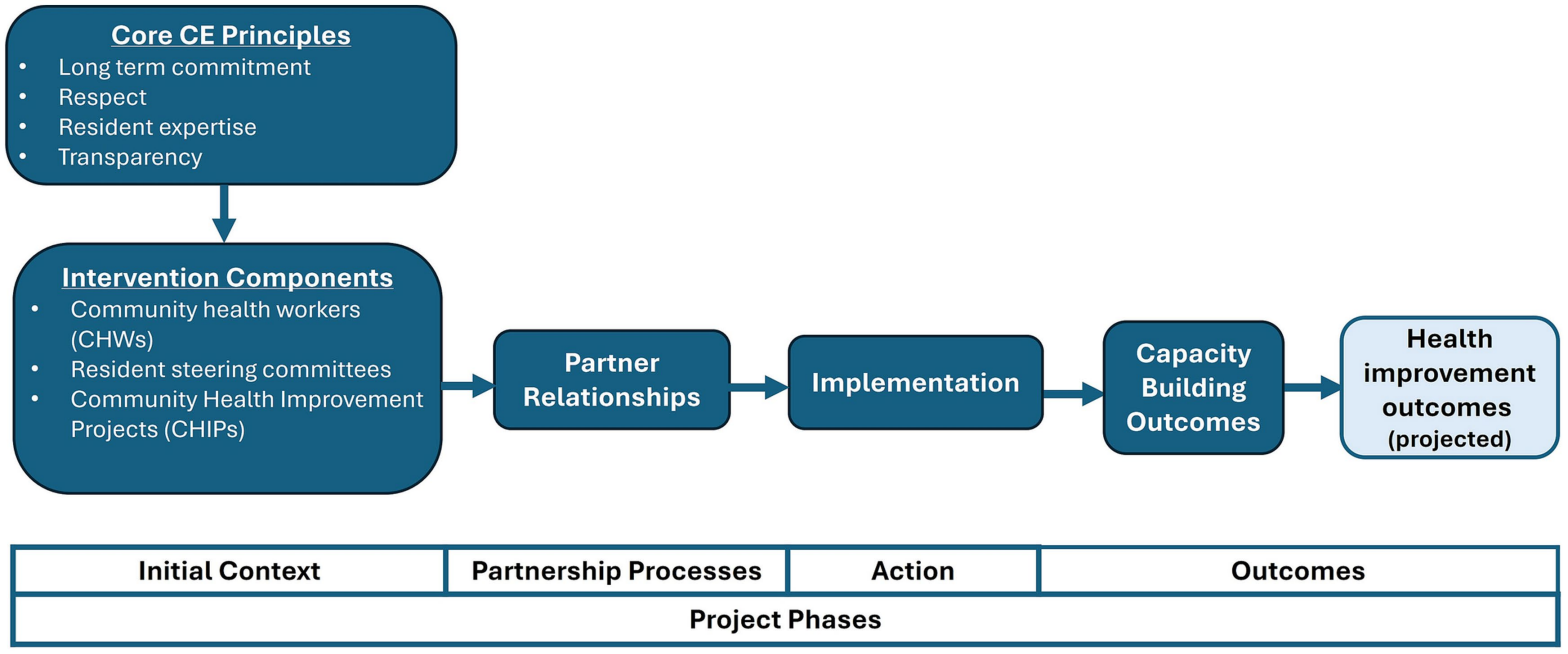
Conceptual model of how DIP-IN principles and intervention components drive outcomes, across project phases, adapted from Oetzel and colleagues’ CBPR conceptual model (16).

The DIP-IN CE approach is grounded in core principles including long-term commitment, respect, valuing resident expertise, and transparency. The approach is asset-based, drawing on partners’ existing strengths and capacities. Figure 1 offers a conceptual model illustrating how principles and multi-level intervention components drive capacity building and improved health outcomes across the project’s phases.

The backbone organization, (e.g., the “DIP-IN team”) leads partnership coordination, communication, and evaluation. The team includes the project lead, evaluators, administrative staff, and project managers assigned to each community. The team is based at an urban academic institution located in downtown Indianapolis with a strong history of community partnerships and addressing health equity. DIP-IN is supported by funding from a health industry organization, through funds allocated toward global health programs in resource-limited settings. This project does not include funding for medical treatment, and the funder is not involved in the day-to-day project activities. Decision-making on all aspects of the project is in the hands of the project team and partners. Project partners, including residents, are active participants as authors or contributors in many dissemination efforts aimed at academic, practitioner, and lay audiences (24–26, 28, 29, 36–38).

Health system community health workers (hCHWs) focus on tertiary prevention, supporting high-risk individuals with diabetes through home visits. They are managed by our public hospital partner and based in Federally Qualified Health Centers (FQHCs) with workflows that integrate them into the care teams. Neighborhood community health workers (nCHWs) are embedded in community-based organizations and address secondary prevention by raising awareness of diabetes risk factors, promoting screenings, and providing educational outreach. They also meet with local organizations, building capacity by strengthening the network of community resources and support systems.

To advance primary prevention and build community capacity, there is a close relationship between the backbone organization and resident leaders. For over 7 years, residents have been actively engaged, driving portions of its design. Their continued commitment suggests a trusting relationship with DIP-IN. In the first year, resident steering committees (SCs) were established in each of three DIP-IN communities. Members include leaders of neighborhood associations and engaged residents, with an intention to reflect community diversity. Monthly meetings are facilitated by project managers, employees of the backbone organization who liaise between the committee and the community to strengthen networks around primary prevention goals.

Unlike advisory boards that merely provide input, SCs have decision-making authority, which we view as critical for building trust and realizing outcomes. The SCs manage budgets of approximately $100,000 per year to fund evidence-based community health improvement projects (CHIPs) focusing on four primary prevention areas (e.g., healthy food access, physical activity, stress, social connections). When combined, smaller interventions like CHIPs can impact population-level health outcomes within target communities (39). Organizations or individuals submit and present CHIP proposals to the SC for consideration and vote. Oftentimes, CHIP leaders are invited by SC members or project managers. CHIP leaders live and/or work within the DIP-IN communities, providing health resources *and* economic investment back into the community. Although CHWs, project managers, and other partners attend meetings, only residents vote. CHIPs remain accountable to SCs through regular progress reporting and work with the backbone organization evaluation team to build organizational evaluation skills. Examples of CHIPs funded include community gardens (healthy food access), fitness classes (physical activity), yoga (stress), and community dinner series (social connections), with some addressing multiple focus areas. For additional examples of CHIPs and key outcomes measured, see our manuscript on the overall DIP-IN design and evaluation (24).

### Study design

This qualitative study explores capacity building through partner interviews. We contextualize qualitative findings with process measures to give the reader a better idea of the scope of how capacity building was conceptualized and evaluated. Although process measures can demonstrate *if* capacity was built, we rely on interviews with partners to understand *how* and *why* it has occurred, through the voices of those directly involved.

#### Positionality

The co-authors bring diverse perspectives shaped by professional expertise and lived experience, and they share a commitment to addressing health equity through collaboration with communities. Authors 1, 2, 3 & 5 are members of the backbone organization. They have over 25 years of community engaged research experience that spans a range of socioeconomic conditions. While all have lived in Indianapolis, none reside in DIP-IN partner communities. Author 4 is a resident steering committee member and CHIP leader.

#### Process measures

A set of process measures are used to monitor implementation of the three main prevention aims and the capacity building aim. Data are collected in an ongoing manner using REDCap electronic data capture tools hosted at Indiana University (40). The tools were set up by members of the backbone organization, with some of the data entered by CHWs and project managers. CHIP proposals and progress reports are completed by CHIP leaders, and voting forms are completed by SC members. This subset of process measures are indicators of capacity building: (1) Number of individuals who have served as resident steering committee members; (2) Number of CHIPs reviewed and approved by SCs; (3) Number of DIP-IN person encounters associated with CHIPs; and (4) Number of organizations that a DIP-IN project manager or CHW met with for purposes of building the network of community resources and supports. (Each organization is counted only once for each community, but in some cases may be counted in more than one community). For a detailed explanation of how the other aims are assessed and integrated into a comprehensive view of the project’s impact, please refer to our manuscript on project design (24).

#### Partner interviews

Rather than studying capacity building among participants who are the recipients of intervention components (e.g., CHW clients, CHIP participants), this study assessed the project’s impact on individual and organizational participants (e.g., “partners”) who support the components. We conducted 60-minute semi-structured, one-on-one program evaluation interviews (*n* = 28) with DIP-IN partners. Eligible participants were adults who had collaborated with DIP-IN to advance project aims at any point during implementation, had been involved for at least 2 years, and represented four distinct roles (Table 1). All 28 partners contacted agreed to participate. Interviews were conducted in person or via Zoom between 2022 and 2024 (project years 5–7). Each individual was eligible for one interview per year, and completing one interview fulfilled participation requirements for the study. The data presented here reflect the first and only interview for all participants. Each participant received a $10 gift card funded by the study as a token of appreciation. All interviews were conducted by Author 1.

**Table 1:** Interview participants by partner role in DIP-IN community-engaged health equity initiative.

Partner role	Includes	Involvement with intervention components	#
Resident steering committee members	Resident leaders from all three DIP-IN communities	Vote on CHIP proposals Control SC budget Review CHIP progress reports	7
Project staff	Project managers, CHWs	Facilitate SC meetings but do not vote Liaise between SC, CHIPs, community, and key organizational partners Build networks of community organizations	5
CHIP leaders	Leaders of Community Health Improvement Projects (CHIPs)	Receive proposal feedback and funding through SC mechanism Report progress to SC CHIPs focus on four primary prevention areas (e.g., healthy food access, physical activity, stress, social connections)	5
Key organizational partners	Representatives from organizations in academia (backbone organization), health industry (funder), public hospital system, county health department, community development	Involved in design and planning Oversee implementation of project components (e.g., CHWs) Connect to SCs indirectly via backbone organization	*11
Total			28

*11 participants represent 5 organizations.

The interview guide covered three areas: (1) partner and community context, (2) DIP-IN experiences and community impact, and (3) individual and organizational impacts (see Appendix for Interview Guide). We did not pilot this specific interview guide; however, we adapted a similar guide that had been developed and used in multiple studies conducted by Author 1 on participants’ experiences in health equity initiatives. This reflective interview approach supports participants in making sense of past experiences, rather than simply describing them (41, 42). As such, participants tend to highlight the most impactful, meaningful aspects of experience, which speak more to the long-term impacts of interest when studying capacity building. Interviews were audio recorded and transcribed. Interview procedures were deemed exempt by the Indiana University Institutional Review Board.

### Analytic procedures

Each transcript was reduced to a one-page summary using a template based on the interview guide (43). Summaries then supported consensus-making among co-authors, with full transcripts consulted as needed for additional detail or context. To examine capacity-building processes, we focused on summaries of partners who are most actively involved with community decision-making, resident SC members and project staff (*n* = 12). We identified sections of summaries corresponding to capacity building processes as defined above (e.g., CHIP proposal process, participation in SCs) and used tools of grounded theory (44) to code and categorize responses as facilitators or barriers of those processes.

To evaluate capacity-building outcomes, we included summaries of all interviews (*n* = 28), focusing on sections about personal and organizational impacts of participating in DIP-IN. Outcomes were classified as capacity building if they reflected strengthened skills, resources and systems in a way that enhanced abilities to achieve and sustain project goals. Informed by the *Guide to Evaluating Collective Impact* (14), we coded and categorized outcomes by level and participant role (Table 2). Rich examples illustrate DIP-IN’s contributions to outcomes.

**Table 2:** DIP-IN capacity building outcomes by level (individual and organizational) and partner role.

Capacity Building Outcomes	Partner Role*				Representative evidence from partner interviews
	SC	Staff	CHIPs	Key Orgs	
Individual – level capacity building outcomes					
Leadership and professional development	X	X	X	X	**Community leadership:** Gained “grass roots” knowledge about what’s happening in the community (SC member) DIP-IN has “opened up doors” for becoming a better community leader (CHIP leader, social connections) **Expectations of CE:** “…every single community organization, should be run the same way” (SC member) **Professional leadership:** “DIP-IN has actually made me a better organizer, has made me a better community builder, it’s made me a better manager, and a better leader.” (Staff, Project manager) “DIP-IN has changed my approach in how I lead people because I listen to people more than I talk…I defer to my team and let them lead.” (Key org, health industry/funder)
Organizational – level capacity building outcomes					
Created new staffing structures and workflows			X	X	**Clinical CHW staffing structures:** “DIP-IN is sustainable workflow that works…” (Key org, hospital system) **New position dedicated to health:** “So our involvement with DIP-IN has really caused us to take a deep dive at building up our health and wellness aspect of our organization” (CHIP leader, social connections) **Neighborhood CHW staff structures:** DIP-IN helped them “further refine” existing CHW / community builder role and budget for full-time position(s). (Key org, Community development)
Increased capacity to collect, use, & share data			X	X	**Developed evaluation surveys with DIP-IN:** “The surveys have been a good way for us to get a better understanding of…the voice of the people who actually attend…” (CHIP leader, healthy food access) **CHW evaluation structures:** “We have learned a lot from being a part of DIP-IN, in terms of the metrics that we evaluate and the activities and the workflow the CHWs do.” (Key org, Hospital system). **Data sharing between county health department and hospital system:** “DIP-IN increased some of our interactions…and we all serve the same group, so it strengthened the relationship.” (Key org, Hospital system)
Obtained additional funds			X	X	**Allowed partners to demonstrate their experience:** “I’m in the community now already doing the work that I’m proposing. It’s not theoretical. I think that went a long way…” (CHIP leader, physical activity) **DIP-IN as a partner.** “Having DIP-IN as a partner certainly helps with that trust factor with other funders.” (CHIP leader, social connections)
Allocated resources to DIP-IN communities			X	X	**Increased programming in DIP-IN areas:** “I feel like that gives us a bigger impact than just the planned nutrition education class.” (CHIP leader, healthy food access) **Allocated resources to DIP-IN area schools:** “We are providing backpacks and school supplies to every student in that school.” (Key org, health industry / funder)

*SC, Steering Committee members; Staff, Individuals employed through DIP-IN funds; CHIPs, Leaders of community health improvement projects that received DIP-IN funding; Key Orgs, Representatives of city level organizations that have been involved with DIP-IN.

X = At least one partner of that role described the outcome.

Multiple strategies enhance the trustworthiness and transferability of the findings. Participants from diverse partner roles represent diverse capacity-building experiences. While all interpretations were grounded in the data, Author 1 and 5’s closer engagement with partners helped contextualize findings, complemented by the more distanced perspectives of Authors 2 and 3. We intentionally addressed both positive and negative aspects of experience by attending to barriers in the interview guide and analysis. Findings are supported with direct quotes, and detailed descriptions of the project context allow readers to determine adaptability in their context.

## Results

We first explore capacity-building processes through process measures and the perspectives of resident SC members and staff which support intervention components. Then, we describe capacity building outcomes from the perspectives of all partners, attending to the influence of DIP-IN. In each section, we describe the overall patterns of the findings, followed by illustrative examples and interpretive commentary.

### CE processes

#### Capacity-building process measures

To contextualize interviews about capacity-building, we report on related process measures for the period from Year 2 Quarter 1 to Year 7 Quarter 3 – a total of 23 quarters. In that period, 57 residents served as members of one of the three SCs. These SCs reviewed 98 proposals for CHIPs, approving 74 of them. With the understanding that the same individuals may have interacted with CHIPs more than once, we estimate over 46,000 participant encounters associated with these 74 projects, for an average of 622 encounters per project. Furthermore, DIP-IN project managers and neighborhood CHWs met with 456 organizations for the purposes of building a network of community resources and supports. This is an average of 152 organizations unique to each community; an organization is counted only once per community though multiple meetings may occur. These meetings often lead to CHIP proposals and increased collaboration.

#### Experiences of capacity-building processes

While process measures indicate that capacity is being built, interviews shed light on *how* these same processes work, from the perspectives of those most involved (SC members and project staff). Overall, they reported positive experiences with DIP-IN processes, compared to less favorable experiences with other initiatives. Residents explained the contrast in terms of how they were treated—with DIP-IN truly embodying its core CE principles. This suggests CE principles are facilitators of long-term engagement and capacity building. Below we share partner reflections on DIP-IN experiences, organized by CE principles, followed by a summary of barriers to capacity building.

#### Commitment

DIP-IN engaged residents throughout the project, not just the beginning. One resident recalled a leader of a different project asking for resident input up front but not further “down the line.” Another resident described the importance of having a project with a longer timeline. She said, if a project is not at least 3 to 5 years, “we do not need it at all in our community.” Initially funded for 5 years, DIP-IN was later extended.

#### Respect

Residents expressed initial distrust of DIP-IN’s intentions, referencing previous groups in the area with a “savior mentality” or those that aimed to “mine” the community. One noted some other groups just want a “check mark that they have worked in a Black community,” while another described other projects as being “shoved down your throat.” Residents saw DIP-IN as different—one noted it “did it differently” by listening first. Similarly, a resident said that before DIP-IN, she had never experienced a listening-first approach. Another resident said his involvement with DIP-IN fit with his own, larger mission to “push neighborhood empowerment to be able to control our own destiny.” Staff echoed this, with a former CHW explaining that DIP-IN trusted residents “to know for themselves what could work,” rather than acting like outside experts “here to save the day.”

#### Value of resident expertise

Residents described DIP-IN as a space that genuinely supports “resident-driven” decision-making, and “leveling the playing field” with partners, including funders. One resident shared she never felt “belittled” or “beneath” funders, which gave her confidence. She contrasted DIP-IN with “surface level” CE, where neighborhood voices are invited but not truly factored into decisions. A former CHW affirmed this, saying it is vital to listen to the SC because “their voices are being heard, and they matter.” Honorariums and budget control reinforced their value—one resident noted, “How many not for profits actually have a funding purse?… now you have the ability to hold the purse strings and pick and choose what it is in your neighborhood…”

#### Transparency

Residents were initially skeptical about whether DIP-IN would truly benefit them or be transparent with outcomes. In contrast to DIP-IN, one resident criticized other groups as “inspirational” but not “transformational,” praising DIP-IN as a rare exception to those that “claim they are doing good for the people but are really doing good for themselves.” He emphasized, “every dollar that’s invested in is actually something that’s going to help the residents.”

#### Challenges with group dynamics and inclusion

DIP-IN SCs unite neighborhood groups with distinct histories, which can challenge cohesion. One resident shared, “I’d love to see us pull together more… more cohesive, a better relationship and more trust in one another.” Committees also faced difficulties in recruiting members who reflect the community’s diversity. Residents and staff noted the need to engage more Hispanic residents, men, and youth. As one resident put it, “We need to be able to be neighbors to them… they experience the same issues we do.” A former CHW added, “I do not think it was a diverse enough sampling… to be more impactful.”

#### Challenges with adaptation and momentum

One resident reflected on the need to “adjust, reorient, and re-acclimate” amid shifting circumstances like COVID and member turnover. Early on, the “train” of DIP-IN was “slow to start moving,” as one member put it. Now, it is “picking up passengers and delivering things.” Staff echoed this, noting difficulties in how to “move” and prioritizing projects, perceiving residents as focusing on marketing / visibility rather than impact and sustainability. One CHW said, “as beautiful as it is to be community centered and neighbor centered, in a way, also made it very, messy” and challenging to get things done.

Two former neighborhood CHWs felt discouraged by not knowing if they were making an impact and desired “tangible” results. For one, this contributed to “burn out” in a job they were passionate about. They also cited the toll of taking on additional responsibilities as a CHIP leader. Another CHW wished for more resources and training for the complex role. However, she realized later that the DIP-IN lead was “giving us an opportunity to build it.”

### Capacity-building outcomes

Findings from interviews demonstrate a range of capacity-building outcomes related to DIP-IN, across partners and at multiple levels (see Table 2). From an individual perspective, DIP-IN supported leadership development.

#### The SC built on existing leadership capacities of resident leaders

DIP-IN leadership development carried over to other community activities. One resident, president of her neighborhood association, said she developed “true leadership” as she became someone “in the mix” at the grassroots level. Another said, “you guys have enlightened me and set a fire up under me to do more.” While the SC is itself a leadership role, five of seven residents interviewed also became CHIP leaders. One SC elected chairs to lead meetings, a role fulfilled by project staff in the other two committees.

Residents shared their leadership and expertise beyond the SC. Some presented at a national leadership conference, where other communities were “very interested” in replicating DIP-IN. Communities hosted international employees from DIP-IN’s funding organization who sought to learn from residents. One said, “They were very impressed with the community… we have international footprints.”

#### DIP-IN changed expectations of CE initiatives

One SC resident said, “every single community organization should be run the same way,” referring to resident decision-making. Another emphasized, “Funders can make things move… it’s up to us as residents to get them to want to do it.” She also warned that future efforts from different groups might get “watered” down compared to DIP-IN.

#### Partners leveraged DIP-IN CE experiences within their career trajectories

One resident SC member became a DIP-IN project manager and is now a director in city government. He views the world through a “health lens” and follows the “DIP-IN way of doing things,” prioritizing community voices for transformative change. A former CHW now supervises similar staff, at the city-level. DIP-IN “fueled a fire” to address health disparities through community work. A representative of the funding organization had a significant leadership shift. She adopted DIP-IN’s shared decision-making approach and now prioritizes “listening and deferring” to staff. Learning about local health disparities was a “wakeup call” and remains central to her professional “why.”

#### Organizational capacity building

Participants described a range of organizational capacity-building outcomes (Table 2). We explore DIP-IN’s role through the perspectives of three organizations, differing in terms of size, existing capacities, and pathways to outcomes. We highlight ways DIP-IN’s impact on *organizations* might have broader implications for *community* capacity building and even systemic transformations.

#### Community-based organization, community dinners CHIP

The Sunday Suppers community dinner series has been funded annually in one SC since 2022, growing significantly and attracting additional funders. It has sparked indirect, ripple effects including improvements in the immediate area, suggesting a broader community impact. According to the project lead, the relationship with DIP-IN provided a “trust factor” for securing additional funding. DIP-IN influenced her to hire staff dedicated to health and wellness. She stated, “DIP-IN is helping us build a little bit of capacity… so we can focus on the health disparities that are really prevalent in our community and what our part is in trying to combat that.”

#### County health department, nutrition education CHIP

Since 2022, the nutrition education services department has received annual CHIP funding from all three SCs, expanding access to existing programming. Their director reflected, DIP-IN “made it easy” by having already “set up infrastructures in the communities.” SCs have guided program locations, and the DIP-IN evaluation team assisted in designing a post-class survey. A staff member noted, “The surveys have been a good way for us to get a better understanding of…the voice of the people, what did they want?” Due to the partnership, the department has allocated more resources to DIP-IN areas. For this CHIP and others, DIP-IN funding ensured a consistent presence in the community, demonstrating long-term commitment and informing ongoing improvements that supported better health outcomes.

#### Public hospital system, key organizational partner

The public hospital system, which manages health system CHWs, described how DIP-IN strengthened staffing structures and workflows. The CHW supervisor said, “DIP-IN is sustainable workflow that works… you know how often you should engage a patient, how often (you) should be following up.” Structures have been adapted for other projects, including adapting the model for a successful CDC grant application. They also noted improved data sharing with the health department—a systems-level impact that reinforces CE infrastructure.

## Discussion

To address health disparities, community engagement (CE) approaches meaningfully and actively partner with affected communities for the long term. However, when evaluations focus solely on long-term health outcomes, CE efforts may seem ineffective due to the time required for measurable change. Intermediate outcomes, like capacity building, can be used to assess progress toward ultimate health aims. This study evaluated capacity building in the multi-year DIP-IN CE health equity initiative through the perspectives of project partners who support intervention components, including resident members of steering committees, CHWs, funders, project managers, and key organizational and CHIP partners.

Findings suggest that DIP-IN has enhanced partners’ capacities to achieve and sustain project goals, with outcomes varying by role (Figure 1). Capacity-building outcomes include individual leadership development and organizational impacts on staffing structures and workflows, obtaining funding, data practices, and the prioritization of DIP-IN communities. Resident leaders and staff involved in multiple CE structures and processes reported significant leadership and professional growth facilitated by DIP-IN principles of long-term commitment, respect, and valuing resident expertise. Some staff chose to shift career paths to make an even greater impact on health equity, championing the resident-driven DIP-IN CE approach. CHIP leaders and organizations that oversee CE structures reported changes with community and systems level implications. Partners reported process barriers, including group cohesion, inclusive representation, adapting to change, sustaining momentum, and supporting staff in complex roles.

### Implications for CE practice

Findings emphasize the importance of long-term resident-driven CE (2, 11), where CE principles are intentionally integrated across multiple project components. Without a deliberate approach even well-principled efforts can fall short or harm relationships. For example, without a formal voting system, it would be hard to uphold the principle of valuing resident expertise within a DIP-IN resident steering committee. Implementing a formal voting process helped us “stick to our principles,” and foster relationships, a key mechanism linking components of CE initiatives to capacity building (Figure 1).

Although we did not ask participants explicitly about the role of trust, resident leaders contrasted positive DIP-IN experiences with negative, distrustful encounters with other initiatives - an experience all too common among members of marginalized communities. Residents’ reflections aligned with many of Principles of Trustworthiness put forth by the AAMC Center for Health Justice including intentionality, respect, humility, and transparency (45). Findings underscore the foundational role of trust in CE, where it can serve as a precondition, mechanism, or outcome (2, 7, 10). Building trust begins with sharing power: DIP-IN demonstrated this by giving residents control over real budgets and decisions, showing they were leading, not just being consulted. Projects must also be transparent about goals, resources, and impact, as communities can easily distinguish between surface-level or “one off” efforts and those aimed at lasting change. Investment should include leadership development and organizational capacity building. Author 4, a resident leader, emphasized that building trust goes beyond simply attending meetings, it is earned through consistent presence in communities.

For organizational partners, capacity building also rested on long-term trusting relationships, centered around shared goals for community health. While many CHIP leaders have limited direct contact with SCs, they benefited from collaboration with the backbone organization, especially its evaluation team. DIP-IN also fostered collaboration from multi-sector partners around local health disparities—laying the groundwork for broader CE infrastructure. Two central Indiana health systems adapted the DIP-IN CHW model which includes both health system and neighborhood CHWs, and one received a CDC grant to expand its CHW workforce. Additionally, the CHW model was adapted by the Indianapolis Health Equity Access Outreach & Treatment (iHEART) Collaborative to address cardiovascular disease (46). Multiple researchers and practitioners have approached DIP-IN for advice and introductions to community partners, resulting in resident SC members serving on advisory boards for other projects, helping to broaden the reach and impact of CE efforts.

### Contribution to CE research

This study advances understanding of how community engagement (CE) influences multi-level capacity building (12, 14–19) by examining perspectives from partners in diverse roles (31–33). While DIP-IN process measures confirmed capacity gains, partner interviews uncovered underlying drivers—particularly the strength of relationships—that standard metrics could not capture. To support future evaluations, we share three practical tools that can be adapted to other contexts including a conceptual model, interview protocol, and outcomes table. We recommend that capacity building be prioritized in CE evaluations—as an outcome, an indicator of sustainability, and an intermediate step toward long-term health improvements. Furthermore, exploring how different CE approaches shape capacity building can refine strategies for equitable, lasting health outcomes.

### Limitations

While reflective interviews may lend themselves to more subjective interpretations, they support participants in making sense of past experiences rather than simply recalling them (41). Participants often emphasize the most significant impacts, which suggest sustainability beyond the project. The more objective, quantitative measures of capacity building contextualize interview findings. Although this study presents findings from one-time interviews, studies assessing capacity building at multiple time points could assess if / how impacts develop and endure over time, particularly after a project has ended. In this study, we used length of involvement in the project as sampling criteria (e.g., at least 2 years). Although we sampled participants with at least 2 years of involvement, shorter-term participants may also have impactful experiences.

### Recommendations for CE funders and organizational partners

This study highlights the need for long-term, flexible funding for meaningful and effective CE. Building trust and relationships needed to affect lasting change takes time. Our funder, Eli Lilly and Company, recognized this by committing to a duration rarely seen in CE work. However, long-term CE of this nature is not always feasible. Even when every group involved—including residents—is dedicated to sustained CE, funding remains a major obstacle. Viewing CE as part of a broader network—rather than isolated initiatives —can enable creative solutions, such as coordinating shorter, sequential or simultaneous efforts within the same community. As mentioned above, DIP-IN coordinates with multiple Indianapolis-area CE health equity initiatives, including some led by DIP-IN organizational partners. Coordinated or not, it is important to remember that trust (distrust) in one initiative has implications for others.

Finally, this kind of CE calls for reimagining norms for health equity interventions involving community. Funders and other partners must give up control - trusting residents to lead. The complexity of this approach demands evaluation methods that are adaptive, ongoing, and often unfamiliar to those trained in fixed, predetermined measures (47–49). Robust and flexible qualitative methods play a critical role. Communication tools are needed for conveying relevant outcomes like capacity building to diverse audiences, broadening definitions of intervention efficacy.

## Conclusion

A quote from a SC member inspired this paper’s title and encapsulates the impact of DIP-IN’s CE approach. She hoped the SC and community could one day say they were “part of change.” For many, DIP-IN fostered a deep sense of ownership. Being “part of change” means contributing to something bigger and leaving a legacy. The phrase also captures the broader impact of the project and its evaluation. CE unites residents, staff, and organizations—each “part of change”—to build capacity and improve health. Though long-term health outcomes take time to come to fruition, capacity-building results can be shared early and often, so everyone can see their role in the progress toward health equity.

## Data Availability

The datasets presented in this article are not readily available because in our IRB, we indicated we would not share interview transcripts publicly. Requests to access the datasets should be directed to celrnich@iu.edu.
