# Supplementary material for: Being part of change: partner perspectives on capacity building in a long-term community-engaged health equity initiative
Source: Front Public Health. 2026 Jan 29;13:1633625. doi: 10.3389/fpubh.2025.1633625 (PMC12894333; doi:10.3389/fpubh.2025.1633625)
Supplement: Supplementary file 1 [file Data_Sheet_1.PDF]

Appendix: Sample Interview Questions
Diabetes Impact Project – Indianapolis Neighborhoods

Individual and Community Context

1. When/how did you first become involved with DIP-IN?
   - ❑ What was your first impression?
2. Describe your activities in the community prior to DIP-IN?
3. What initiatives involving residents of the community were happening around that time?
4. How did you see the project fitting into the community, given what you know about its history and culture?
   - ❑ What opportunities and threats to success were you aware of at the time?

Project Experiences and Community Impact

5. What role(s) have you had with DIP-IN?
6. How would you describe DIP-IN to someone who doesn't know about the project?
7. How does DIP-IN's approach compare to other community-engaged initiatives you and/or your organization been a part of or aware of?
8. What value do you think community health workers bring to overall community health and wellbeing?
9. What unique value do you think the steering committees bring to overall health and well-being?
10. How has the DIP-IN project as a whole impacted the community, to this point?
    - ❑ Can you think of a time when you were concerned that DIP-IN would not make a meaningful difference in the neighborhood?
    - ❑ Can you identify a point in time where DIP-IN partners and programs were starting to gain momentum?
11. What would it look like for DIP-IN to be "sustainable?" in the future?
12. What is your long-term vision for health and wellbeing in the?

Individual and Organizational Impact

13. What, if any, changes have you made to your organization, or its priorities based on your involvement with DIP-IN? These could be changes which are planned or already being implemented.
14. What have you taken away personally, professionally, or as a leader from your participation in DIP-IN, to this point?
15. What have you learned from being involved in DIP-IN?
16. Please tell me about a success you had in DIP-IN? What are you most proud of helping accomplish?
17. Please tell me about a challenge you had or have in DIP-IN?
18. What keeps you involved with the project?
19. How can DIP-IN support you in your role?
20. Is there anything else you would like to add about your experiences in DIP-IN?
